# Evaluation of Peripartum Hysterectomy in a Tertiary Care Unit and Its Effect on Patients' Long-Term Physical and Mental Wellbeing: Quest Is Not Over When You Save the Life

**DOI:** 10.1155/2021/5720264

**Published:** 2021-02-17

**Authors:** P. D. M. Pathiraja, Asanka Jayawardane

**Affiliations:** ^1^Obstetrics and Gynaecology, Ministry of Health, Colombo, Sri Lanka; ^2^Obstetrics and Gynecology, University of Colombo, Colombo, Sri Lanka

## Abstract

**Objectives:**

Peripartum hysterectomy can be performed as an elective procedure or as a life-saving emergency procedure in obstetrics. It is associated with significant maternal morbidity and mortality. We report peripartum hysterectomies done during the study period in a tertiary referral centre, Colombo, Sri Lanka. *Methodology*. We collected data on all severe acute maternal morbidity and mortality events (SAMM) from June 01, 2014, to June 01, 2015, at De Soysa Hospital for Women (DSHW). We invited all women who underwent PPH to complete the 36-Item Short Form Health Survey questionnaire (SF-36) before hospital discharge and at six months after the hysterectomy date to assess their general and mental health before and after surgery. Focus group discussions (FGD) were used to further evaluate the patient experience and to identify service delivery improvements.

**Results:**

There were eleven peripartum hysterectomies done during the study period for 7160 deliveries. None were primigravida. Median age and gestation were 36 years and 37 weeks, respectively. The commonest indication for peripartum hysterectomy was a morbidly adherent placenta (seven). Nine of the deliveries were elective lower-segment caesarean section and two were vaginal deliveries. Four emergency peripartum hysterectomies were done for primary postpartum haemorrhage (PPH) and two for secondary PPH. All patients required intensive care and there were no maternal deaths. The analysis of SF-36 data revealed that all patients suffered a significant reduction in the quality of life at six months after the surgery. FGD highlighted that most patients needed further counselling and support to improve their physical, psychological, and social wellbeing. Some of the patients were willing to share their experience on voluntary basis to help those undergoing peripartum hysterectomies in the future.

**Conclusion:**

Peripartum hysterectomy is an important life-saving procedure associated with severe maternal morbidity and mortality. This study reveals that the physical, psychological, and social adverse effects would remain in the long term.

## 1. Introduction

Hysterectomy following childbirth (peripartum hysterectomy) to save the mother's life includes emergency hysterectomy performed following vaginal delivery or caesarean section and elective cesarean hysterectomy for indications including morbidly adherent placenta (MAP). MAP comprises placenta accreta, increta, and percreta. Peripartum hysterectomy was first described by Porro, and it was used to prevent deaths due to severe PPH [1]. Although it is a rare event in obstetric care, it is an indicator of severe acute maternal morbidity (SAMM) [2].

The reported incidence of peripartum hysterectomy varies from 0.2 to 8 per 1000 deliveries [3–5]. Although rare, the incidence of peripartum hysterectomy is increasing. In both developed and developing countries, the increase in the incidence of peripartum hysterectomy has been attributed to the increasing caesarean section rates, giving rise to a parallel rise in the incidence of placenta praevia and MAP which has increased 10-fold over the past few decades [6]. Additional factors for peripartum hysterectomy within developing countries include delay in transfer to tertiary care centres, lack of one-to-one care in labour room, some religious and cultural beliefs, poor antenatal care, poverty, and lack of health facilities [7].

The decision to perform a peripartum hysterectomy on a young woman, especially with low parity, is very challenging. However, a timely decision can make the difference between life and death. For the woman who survives, peripartum hysterectomy is an event which saved her life. However, many women following peripartum hysterectomy have long-term physical, psychological, and social morbidity. Common physical complaints by women following peripartum hysterectomy include chronic pelvic pain, dyspareunia, and general body weakness.

Psychological sequelae are also described following peripartum hysterectomy with women suffering from posttraumatic stress disorder, depression, insomnia, and personality disorders [6–8]. The social problems these women faced include relationship and financial difficulties. It seems that these problems persist in the long term.

There are limited explorations into the recognition and management of such complications. In Sri Lankan context, the problems these women face in readjusting to a normal life have never been previously explored.

It is important to study the postsurgical recovery to ensure women receive relevant and adequate care and support. We aimed to study the physical, psychological, and social problems women faced following a peripartum hysterectomy.

## 2. Materials and Methods

### 2.1. Study Settings

This is an institution-based, observational study, conducted at De Soyza Hospital for Women (DSHW), Colombo, Sri Lanka, over one year. DSHW is the first maternity hospital in Sri Lanka and the second oldest maternity hospital in Asia. It serves 8,000–10,000 expectant mothers annually and receives high-risk obstetric referrals from all over the country.

### 2.2. Study Population

This study included all women who underwent peripartum hysterectomy (both elective and emergency) at DSHW from June 01, 2014, till June 01, 2015.

Informed written consent was obtained from all participants before discharge from the hospital. Information was available in Sinhala, Tamil, and English. Opportunities to ask questions and register complaints were provided in the information sheet and were also verbally explained at the time of consent. Relevant contact details were included in the information sheet.

### 2.3. Data Collection and Analysis Methods

Patients' records of all who underwent peripartum hysterectomy were analysed for demographic profile, clinical characteristics, and operative notes for indications, intraoperative findings, duration of surgery, blood loss, anesthesia records, duration of stay, and postoperative events. Even though magnetic resonance imaging (MRI) is the gold standard method to diagnose MAP, we used ultrasound scan (USS) for this purpose, as MRI facilities were not freely available during the study period.

We used the 36-Item Short Form Health Survey (SF-36) to assess the patient health and quality of life (HR-QOL), which was already validated to Sinhala and Tamil languages. It consists of 36 items under eight domains, which are the weighted sums of the questions in their section [9, 10]. Each scale is directly transformed into a zero to 100 on the assumption that each question carries equal weight. In SF 36, a high score indicates less disability and a low score indicates more disability.

The first three domains which are physical function, role limitation, and bodily pain measure the patients' physical well being. General mental health, role limitations due to emotional problems, and social functioning are assessed with their mental wellbeing. The two other domains, fatigue and general health perception, assess both physical and emotional aspects of heath. The responses to all these items should be a reflection of the preceding four weeks.

The SF-36 study questionnaire was applied during immediate recovery period to all women who underwent peripartum hysterectomy to recall and fill based on their previous experiences. The same study tool was used for all women at six months after the surgery to compare the results before and after.

Data were analysed using the Statistical Package for Social Sciences (SPSS, version 20). The mean value was used for describing the central tendency of observation for each variable. *p* value of less than 0.05 was regarded as statistically significant.

Focus group discussions (FGD) were used for in-depth assessment of patient concerns and their experiences [11]. Information was gathered through direct interview in a setting most convenient to the patients in hospital premises. There were two FGD comprising six patients and five patients each.

### 2.4. Objectives of the Study

The main objective of the study was to evaluate how the peripartum hysterectomy affects women's health-related quality of life. The study also assessed the prevalence, indications, surgical outcomes, and major complication following peripartum hysterectomy for both the mother and the baby. Focused group discussions were used to evaluate the patients' concerns and how to improve the patients' care delivery in a more user-friendly way.

### 2.5. Ethical Consideration

The research project was approved by the Ethical Review Committee, Faculty of Medicine, University of Colombo (EC-15-122).

Written informed consent was obtained from the patients after explaining the purpose of the study.

### 2.6. Consent

Consent forms were prepared in all three local languages and adequate information provided to women before obtaining informed written consent. All elective caesarian hysterectomy patients consented before the procedure, while emergency group was approached for consent on postoperative period around postoperative day three or four.

## 3. Results

There were eleven hysterectomies (nine at the time of delivery and two in postpartum period) performed during the study period for 7160 deliveries. None were primigravida. Median age was 36 (range 31–36) years and gestation was 37 weeks. Six (70%) patients were more than 35 weeks of gestation at the time of surgery; four (20%) were between 31 and 34 weeks. There were nine live births and two stillbirths (one due to acute fatty liver of pregnancy and the other due to severe fetal growth restriction). The mode of delivery for nine was caesarean section and two vaginal deliveries. Seven patients have had a previous uterine surgery or lower-segment caesarean section (LSCS). Indication for peripartum hysterectomy in this study included MAP (63%), utrine atony (27%), and uterine rupture (9%) (Table 1).

### 3.1. Elective Peripartum Hysterectomy Group

All had hysterectomies due to antenatally diagnosed MAP based on USS. We optimized their haemoglobin level and counselling sessions were arranged before surgery. All had steroids cover (intramuscular dexamethazone 6 mg twice daily for 48 hours) for fetal lung maturation. Classical caesarean with midline laparotomy and peripartum hysterectomy was performed at 35 to 36 weeks under general anesthesia with participating multidisciplinary surgical and anesthetic team. The mean duration of the surgery was 180 minutes.

All elective surgeries were carried out as total hysterectomies. Following the surgery, all patients were transferred to intensive care unit (ICU) for observation. The mean blood loss in the planned category was 660 ml, and the average transfusion requirement was 1.0 packed red cells, and average total hospital stay was eleven days (Table 2). No adverse fetal outcome was seen in the planned group.

### 3.2. Emergency Peripartum Hysterectomy Group

Four women had peripartum hysterectomy due to primary PPH (three for atonic uterus and one due to uterine rupture) and two were due to secondary PPH following failed conservative management of MAP. The latter two were opted for a conservative management where placenta was left in situ after caesarean section and uterus repair. Both received methotrexate to accelerate placental apoptosis. One woman presented with severe secondary PPH after three weeks and received massive blood transfusion and an emergency hysterectomy was performed after resuscitation. The other woman presented with suspected infection and mild bleeding after four weeks. An examination under anesthesia and attempted evacuation of products (ERPC) was performed. She had an emergency hysterectomy due to the placenta being densely adherent to the uterine wall.

Out of the six emergency hysterectomies, four were total and two were subtotal, with mean blood loss of 2033 ml. Transfusion of packed cells was required for all patients (range of 1–9 packed cells) and other blood products were transfused in five patients (Table 2). All patients (four) with atonic uterus were managed according to institutional protocol with medical (oxytocin, ergometrine, tranexamic acid, and misoprostol) and surgical procedures (balloon tamponade methods and brace sutures) before proceeding to hysterectomy. One patient had acute fatty liver of pregnancy (AFLP) prior to delivery.

The most common maternal complication (Table 3) was postpartum pyrexia (28%). Wound infection occurred in 5% of the women. Bladder injury occurred more in the emergency group and all were repaired by urologist and healed completely. There were no maternal deaths, disseminated intravascular coagulation, or bowel injuries in either group.

### 3.3. Assessment of HR-QOL after Surgery

Validated Sinhala and Tamil versions of the SF-36 form were used for evaluation of the physical and mental health of the patients six months after the surgery. Questionnaire was used first during the hospital stay after the surgery (patient recruitment) but before discharge from the hospital for assessing presurgery score and repeated at 6-month follow-up for the postsurgery score.

Out of eight components, five components showed a significant reduction in scores (Table 4). There was a significant increase in postoperative fatigue. Bodily pain showed a slight rise, but not significant. Social functioning and role limitation due to emotional problems showed a slight decline but was not statistically significant.

Further, we noted that 36% of study population suffered from severe dyspareunia six months following surgery, which significantly affected their sexual relationship. It took an average of four months to resume their normal day-to-day activities and at least four to six months to start sexual intercourse after a peripartum hysterectomy.

### 3.4. Focused Group Discussions (FGD)

We would like to elaborate on few points that were highlighted during the FGDs by the participants. All patients expressed that they had undergone immense psychological trauma during this period.

They struggled to maintain the family role after such a major life event. Further, they pointed out the need of a long-term follow-up plan until they achieve full recovery, which includes mental health support and medical therapy when necessary.

“Since I suffered both physically and psychologically and at times felt that I was alone despite having my family around, it would have been beneficial if I was assessed, advised and supported by the medical team. If such a plan could be put in place, supplementing care with home visits would be of great benefit.” (FG1).

Some patients highlighted that they had lost their occupation as a result of long-term absenteeism and even they turned up for work they could not perform at expected standards adversely affecting their financial state.

“This traumatic experience made a huge difference to my life in a very negative manner. I'm still suffering. I lost my job after the surgery due to its consequences—I could not obtain leave from my working place, had to come back to the hospital and get re-admitted and that made it the worst experience of my life.” (FG1).

Also some patients highlighted the benefit of sharing their experiences among the group which would help boosting the confidence of others.

“After realizing that I had a very traumatic and a major life event where my life was saved by the medical team, and looking back very happily at how I faced the consequences of it, I would like to share my experiences with others who might face a similar situation.” (FG2).

## 4. Discussion

Sri Lanka as a developing country achieved remarkable success in reducing maternal mortality rate (MMR) to 30–40 per 100,000 live births, which is a very low rate, compared to other neighboring countries. It has remained static with minimal downward impact over the last decade [12]. While medical disorders in pregnancy (specially the cardiac and respiratory causes) stood at the top of the list, obstetric haemorrhage is the fourth leading cause of MMR. As MMR is just describing the tip of an iceberg, the World Health Organization (WHO) has emphasized on the concept of SAMM [2]. For the women who survive from SAMM, the event contributes significantly and has a major impact on their life. For the health care delivery providers, much importance in quality of care may be gained through reduction of SAMM by strengthening promotive and curative health care delivery [13].

In the current study, the incidence of peripartum hysterectomy of 11/7160 deliveries is higher compared to some other studies [4, 5]. In our study, the most common indications of peripartum hysterectomy were MAP (63%). This high incidence is likely due to DSHW as a tertiary referral centre accepting cases of MAP from all over the country.

Over past few years, the incidence of peripartum hysterectomy has increased and the indications have changed. Globally, most of the hysterectomies were performed because postpartum haemorrhage has been replaced by MAP. Clarke et al. reported atonic uterus in 43% while placenta previa or MAP in 34% cases in 1984 which reversed nine years later to MAP in 45% and atony in 20% [14]. There was a significant difference in the incidence of peripartum hysterectomy following vaginal delivery and LSCS. Even though the incidence of peripartum hysterectomy after vaginal delivery is more constant between European and American studies, the incidence following caesarean section is rising [4, 5]. This would be attributed to the proportion of women with previous LSCS with the concomitant risk of placenta previa and MAP. A meta-analysis based on 98 full-text studies by Juniaux et al. found that the prevalence for the adherent and the invasive grades was 0.5 (95% CI = 0.3–0.36) and 0.3 (95% CI 0.2–0.4) per 1000 births, respectively [15].

Evidence suggests that patients with placenta previa and scarred uterus had a 16% risk of undergoing emergency peripartum hysterectomy compared to 3.6% in patients with unscarred uterus [16]. In the presence of a placenta previa, the risk of placenta accreta was 3%, 11%, 40%, 61%, and 67% for the first, second, third, fourth, and fifth caesarean, respectively [17]. Advanced maternal age, multiparity, and any condition resulting in damage to myometrial tissue also can lead to the development of MAP. We used ultrasound for the diagnosis of MAP as MRI facilities were not freely available. The reported diagnostic sensitivity of ultrasound in MAP is 77–87% and specificity of 96–98%, a positive predictive value of 65–93%, and a negative predictive value of 98% [18]. Jauniaaux et al. evaluate the accuracy of real-time ultrasound in the diagnosis of MAP and the impact of the depth of invasion on patient with low lying placenta and previa. The results found that ultrasound scan is highly sensitive and specific in the prenatal diagnosis of MAP with skilled operators [19].

### 4.1. Lessons Learnt from Surgery

All diagnosed women with MAP were cared for using a multidisciplinary approach (MDT) which included obstetrician, gynaeoncologists, urologists, vascular surgeon, neonatologists, hematologist, anesthetist, and interventional radiologists in planning antenatal care and delivery. The Royal College of Obstetricians and Gynaecologists (RCOG) suggests a six-element care bundle which should be applied in MAP [20–22].

### 4.2. Management of MAP

There are three main options available for the management of MAP:

1. For the delivery of the baby and attempted delivery of the placenta, most of the time the incision is through the placenta site. The majority of women require massive blood transfusion and currently this is not a recommended method.
2. For the delivery of the baby via a uterine incision distant from the placenta, cutting the cord close to insertion site, full repair of the uterus, and conservative management. We managed two patients with the aim of conserving the uterus; unfortunately both had emergency hysterectomies due to secondary postpartum haemorrhage and severe infection. The main drawback is the uncertainty as to the time of onset of secondary bleeding or infection and the need for continued vigilance with 24/7 access to comprehensive tertiary level care. Prophylactic antibiotics may be helpful in the immediate postpartum period to reduce this risk. The current evidence suggests that neither methotrexate nor arterial embolization reduces these risks and are not recommended [23].
3. For the delivery of the baby via uterine incision distant from the placenta, quick repair of the uterus and en-bloc hysterectomy was the preferred method. We performed all elective peripartum hysterectomies around 36 weeks of gestation. This decision should be made jointly with the patient and the team together considering the facilities available for neonatal care. All women were given steroids for fetal lung maturation [24].

For all the elective cases, general anesthesia was used and in the situation of massive postpartum haemorrahge conversion from spinal anesthesia to general anesthesia was considered in other cases. For the elective procedures, epidural catheter was placed preoperatively to cover analgesia postoperatively [25]. Prophylactic antibiotics are indicated, with repeat doses after two hours of surgery or in massive blood loss.

Cross-matched blood and blood products were readily available in anticipation of massive obstetric haemorrhage. The team involved the Haematologist and Transfusion Medicine specialist with the use of thromboelastography (ROTEM) to guide clotting factor replacement [26]. Prophylactic catheter placement of balloon by interventional radiologist for occlusion of internal iliac artery just after delivery of the baby was employed in all elective cases [27]. Prophylactic anticoagulation in these women can be hazardous and the decision was taken on an individual basis after MDT evaluation [28].

The choice of skin incision was made based on the patient's body habitus and previous surgeries in the abdomen. We recommend a high midline vertical incision as suitable for most cases as it provides adequate access for a uterine incision to deliver the fetus avoiding the placenta and subsequent hysterectomy. A classical uterine incision is often transfundal because it avoids the placenta and allows delivery of the fetus. Placental mapping before the surgery is beneficial to position the incision avoiding the placenta. Generally, manual placental removal and oxytocin should be avoided in MAP. After delivery of the fetus, we cut the cord and leave it inside the uterus and use a whip stitch to close the uterus to achieve haemostasis before proceeding with hysterectomy.

We performed subtotal hysterectomy in two emergency situations cases of primary postpartum haemorrhage. All elective surgeries had total hysterectomy. Described proportion of subtotal hysterectomy performed for postpartum haemorrhage ranges from 50% to 80% [14]. The subtotal hysterectomy was associated with lesser blood loss, and therefore less blood transfusions, reduced operating time, and reduced intra- and postoperative complications but it may not be as effective in the management of MAP located in the lower uterus. We used preoperative cystoscopy with placement of ureteral stents to prevent ureteric injuries in some elective surgeries and three-way catheter placed in the bladder to allow irrigation, drainage, and distension of the bladder to help during dissection.

Reported average blood loss at delivery in women with placenta accreta is 3–5 liters [20, 21]. We reported 660 ml in elective procedures and 2033 ml in emergency cases. Maternal mortality with MAP has been reported to be as high as 7% in some reports [29]. There were no maternal deaths in our study.

With experience, we have refined our protocols in managing these difficult cases. Team discussion of the procedure in detail both by junior doctors and consultants with patient briefing for informed consent is done before surgery. All elective peripartum hysterectomies were done with participation of two experienced consultants. We have changed the instruments used for the hysterectomy with experience. Normal hysterectomy clamps (with antislip tooth) cause more tissue damage as tissues are more fragile and carry a higher bleeding risk, and instead of that, we recommend the use of Howard Kelly haemostatic forceps for clamping instead. Compression of the descending aorta reduces massive, uncontrollable bleeding from the uterus. All women were admitted to the ICU for observation but encouraged early mobilization. The team did debrief the women and their family members immediately after the surgery.

Deffieux et al. evaluated the use of intra-abdominal packing for uncontrollable persistent bleeding after peripartum hysterectomy. They found that abdominal packing, used for duration of 24 to 48 hours, is a good option for patient with life-threatening postpartum bleeding after hysterectomy [30]. A study conducted at China among patients with MAP with high-intensity focused ultrasound (HIFU) and uterine artery embolization (UAE) for retained placenta accreta [31]. Results showed that both HIFU and UAE combined with hysteroscopic resection seem to be safe and effective procedures in cases of MAP.

### 4.3. Effect on Women and Their Families Recovering from the Experience

The SF-36 is a standardized questionnaire which has been used in different research settings. This tool consists of 36 items to evaluate under 8 domains. This is the first study to use SF-36 as a tool to HR-QOL after peripartum hysterectomy, although it has been used for abdominal and vaginal hysterectomies in the past. We have given the study questionnaire to all patients who underwent hysterectomy immediately after the surgery to recall and fill it based on their previous experiences. Then, we gave the same tool for all the patients six months after the surgery and compared the results. Therefore presurgery score is a patient reflection of her QOL, taken after the event, and this may have introduced bias. However, this was unavoidable due to the nature of the patient recruitment for the study. This limitation was accepted as we aimed to look at the effects of surgery on individual patients' functioning rather than comparing with a control group of women with in-depth follow-up at the focus group setting.

Both physical functioning and role limitation subscales showed significant deterioration of scores after surgery. These subscales concern about routine day-to-day activities. The bodily pain subscale included items about pain scores and to what extent pain has interfered with day-to-day activities and their occupation. Six months after the surgery, we found no significant difference in bodily pain subscale, suggesting that pain was not a limiting factor for normal activities and work.

The social functioning and mental health subscales included items such as interference with normal social life due to physical and emotional problems and also feelings of nervousness and depression. In our study, major surgical intervention seemed to target the scale in a negative manner. Reduction in physical functioning affected their daily life and made it difficult to cope with work at home. Due to deterioration of role limitation, they were unable to perform their tasks properly and did not engage with full concentration at work, in turn affecting their productivity, self-esteem, and family life.

A prospective cohort study among women with MAP and who underwent caesarean hysterectomy at Utah revealed that ongoing decreased QOL and long-term health issues for up to 3 years following surgery than those undergoing LSCS for other indications [32]. At 36 months, women with MAP who underwent PPH were more likely to report grief, depression, anxiety, and additional surgeries.

Differences of HR-QOL values among emergency and elective categories were not significant with regard to postoperative assessment in six months in this small group of women. However, it will need further exploration with a larger group of patients.

FGD participants revealed that peripartum hysterectomy was a very traumatic event for women, affecting physically and psychologically with consequences on relationships, personal mental health, and in some cases even their employment. This article highlights the need of developing patient peer groups which women found very useful in their recovery process.

## 5. Conclusion

Peripartum hysterectomy is the most dramatic obstetric surgery performed as a last resort in severe postpartum haemorrhage or at risk of major haemorrahge in case of MAP. The indication for peripartum hysterectomy in recent years has changed from uterine atony to MAP.

In-depth analysis of peripartum hysterectomy is important in understanding SAMM, where it allows better resource utilization. SF-36 analysis and FGD gave us a window to concern about the patient and longer follow-up plan to improve their HR-QOL.

Women recovering from peripartum hysterectomy events undergo a different and much prolonged recovery process than we initially expected. Effects on physical, mental, and social functioning need to be documented and communicated by the clinical team for these women to receive the long-term care and help they need. Women, family, primary health providers, employers, and insurance providers need to be educated regarding the prolonged recovery process with realistic expectations for the women to receive much needed support.

Although saving lives is important, a thorough multidisciplinary supportive care for much longer duration is required for these women for a successful recovery process. Policy planners, health insurance providers, and health care teams need to join hands in providing holistic care.

## Figures and Tables

**Table 1:** Indications for peripartum hysterectomy.

Variable		Number
*Planned hysterectomy*	(Elective)	5
Morbidly adherent placenta		5

*Unplanned hysterectomy*	(Emergency)	6
Uterine atony		3
Uterine rupture		1
Morbidly adherent placenta (secondary PPH)		2

**Table 2:** The maternal characteristics compared in the two groups (planned/emergency peripartum hysterectomy).

Variable	Planned group (*n* = 5)	Emergency group (*n* = 6)
Mean blood loss (ml)	660	2033
Average blood transfusion (packed red cells units)	1.0 (0–2)	3.5 (1–9)
Mean ICU stay (days)	4 (1–12)	6 (1–18)
Average hospital stay (days)	11	12
Total hysterectomy	5	4
Subtotal hysterectomy	0	2

ICU: intensive care unit.

**Table 3:** List of surgical complications.

Complication	Planned group	Unplanned group
Postpartum pyrexia	2	3
Bladder injury	1	2
Wound infection	1	1
UTI	1	2
DIC	0	0

UTI: urinary tract infections. DIC: disseminated intravascular coagulations.

**Table 4:** Summary of SF-36 assessment.

Variables	Preoperative mean	Postoperative mean (six months later)	SD	SEM	*t* value	Significance (*p* value)
Physical functioning	100.00	89.82	9.421	2.841	3.584	0.005.
Role limitation	100.00	78.36	32.178	9.702	2.230	0.043
Bodily pain	98.2	95.6	14.752	4.448	6.908	0.091
Social functioning	100.00	93.36	11.509	3.470	1.912	0.085
General mental health	85.45	70.36	14.377	4.335	3.481	0.006
Role limit due to emotional problem	100.00	85.73	32.169	9.699	1.472	0.172
Fatigue	60.45	80.91	13.110	3.953	5.175	0.001
General health perception	83.73	53.73	13.864	4.180	7.177	0.001
Overall						0.001

Significance at *p* < 0.05. SD: standard deviation. SEM: standard error of mean.

## Data Availability

The raw data used to support the findings of this study are available from the corresponding author upon request. The authors will obtain the approval from ethical review committee before handing over all the data if there is any request.

## Abbreviations

SAMM: Severe acute maternal morbidity and mortality events
FGD: Focus group discussions
MAP: Morbidly adherent placenta
PPH: Postpartum haemorrhage
HR-QOL: Health and quality of life
USS: Ultrasound scan
SD: Standard deviation
SEM: Standard error of mean.

## Additional Points

*Strengths*. We were able to collect data from all the patients who underwent peripartum hysterectomy in the study period and did not decline the invitation to participate in the study. We have also analysed both the quantitative and qualitative aspects of the patients' experience. *Deficiencies*. This study has been carried out only in a single unit, within a period of one year. There can be a recall bias as we have collected preoperative data just after the surgery. *Suggestions*. We suggest that this study be replicated in multiple units island-wide to collect more data and to develop standard follow-up plan for patients who underwent major obstetric surgeries and evaluate the need to develop peer group discussions in supporting women experiencing major obstetric surgeries.
